# Integrated multi-omics profiling reveals the ZZZ3/CD70 axis is a super-enhancer-driven regulator of diffuse large B-cell lymphoma cell-natural killer cell interactions

**DOI:** 10.3389/ebm.2024.10155

**Published:** 2024-09-23

**Authors:** Xi Li, Juya Cui, Liao Wang, Caihong Cao, Hu Liu

**Affiliations:** Cancer Center, Shanxi Bethune Hospital, Shanxi Academy of Medical Sciences, Tongji Shanxi Hospital, Third Hospital of Shanxi Medical University, Taiyuan, Shanxi, China

**Keywords:** diffuse large B-cell lymphoma, cell interaction, super-enhancer, natural killer cell, CD70

## Abstract

Tumor immune microenvironment is crucial for diffuse large B-cell lymphoma (DLBCL) development. However, the mechanisms by which super-enhancers (SEs) regulate the interactions between DLBCL cells and tumor-infiltrating immune cells remains largely unknown. This study aimed to investigate the role of SE-controlled genes in regulating the interactions between DLBCL cells and tumor-infiltrating immune cells. Single-cell RNA-seq, bulk RNA-seq and H3K27ac ChIP-seq data were downloaded from the Heidelberg Open Research Data database and Gene Expression Omnibus database. HOMER algorithm and Seurat package in R were used for bioinformatics analysis. Cell proliferation and lactate dehydrogenase (LDH) release was detected by MTS and LDH release assays, respectively. Interaction between B cell cluster and CD8^+^ T cell and NK cell cluster was most obviously enhanced in DLBCL, with CD70-CD27, MIF-CD74/CXCR2 complex, MIF-CD74/CD44 complex and CCL3-CCR5 interactions were significantly increased. NK cell sub-cluster showed the strongest interaction with B cell cluster. ZZZ3 upregulated the transcription of *CD70* by binding to its SE. Silencing CD70 in DOHH2 cells significantly promoted the proliferation of co-cultured NK92 cells and LDH release from DOHH2 cells, which was counteracted by ZZZ3 overexpression in DOHH2 cells. CD70 silencing combined with PD-L1 blockade promoted LDH release from DOHH2 cells co-cultured with NK92 cells. In conclusion, DLBCL cells inhibited the proliferation and killing of infiltrating NK cells by regulating ZZZ3/CD70 axis. Targeting ZZZ3/CD70 axis combined with PD-L1 blockade is expected to be a promising strategy for DLBCL treatment.

## Impact statement

In this study, we found that CD70 was a super-enhancer-controlled gene that was driven by ZZZ3 for transcription in diffuse large B-cell lymphoma cells. The ZZZ3/CD70 axis in diffuse large B-cell lymphoma cells inhibited infiltrating natural killer cell killing and proliferation, thereby promoting immune evasion of diffuse large B-cell lymphoma cells. The ZZZ3/CD70 axis has the potential to be a novel immunotherapy target for diffuse large B-cell lymphoma. Targeting ZZZ3/CD70 axis combined with PD-L1 blockade is expected to be a promising immunotherapeutic strategy for the treatment of diffuse large B-cell lymphoma.

## Introduction

Diffuse large B-cell lymphoma (DLBCL) is the most common B-cell non-Hodgkin lymphoma with highly heterogeneous and aggressiveness [1, 2]. Although therapeutic strategies such as chemotherapy, radiotherapy and immunotherapy have improved the survival of DLBCL patients, the prognosis remains generally dismal for patients developing relapsed or refractory DLBCL [3, 4]. Identification of novel therapeutic targets is essential to improve the outcomes of patients with DLBCL. Understanding the pathogenesis of DLBCL could facilitate the development of novel molecular therapeutic targets.

Interactions between tumor cells and tumor-infiltrating immune cells in the tumor microenvironment (TME) could either induce tumor suppression or promote tumor development [5–7]. For example, ligands on the surface of tumor cells, such as programmed death-ligand 1 (PD-L1), major histocompatibility complex class II (MHC-II), fibrinogen-like protein 1 (FGL1) and galectin-9 (Gal-9), interact with the inhibitory receptors of immune effector cells, such as programmed cell death protein 1 (PD-1), lymphocyte-activation gene 3 (LAG-3) and T cell immunoglobulin and mucin domain 3 (TIM3), to inhibit cytotoxicity of immune cells and promote tumor immune evasion [8–10]. The complex interactions between tumor cells and various tumor-infiltrating immune cells are involved in regulating the immunosuppressive microenvironment of DLBCL [11–13]. Compared with solid tumors, DLBCL has a higher abundance of infiltrating immune cells in the TME [6, 7]. However, interactions between DLBCL cells and tumor-infiltrating immune cells are still not well characterized.

Recently, single-cell RNA sequencing (scRNA-seq) technology has become an important tool for studying the lymphoma microenvironment, revealing the high heterogeneity of tumor cells and their interactions with immune cells. For instance, Roider et al. used scRNA-seq to study DLBCL and shed light on the heterogeneity of nodal B-cell lymphomas, emphasizing its relevance to personalized cancer therapy [14]. Additionally, Steen et al. elucidated the DLBCL microenvironment at a systems-level resolution and identified potential therapeutic targets by integrating multiple scRNA-seq datasets [15]. Despite these systematic insights into lymphoma microenvironment heterogeneity, the epigenetic regulatory mechanisms governing communication between tumor cells and microenvironment cells remain elusive.

Aberrant epigenetic alterations regulate the phenotype of tumor cells, and participate in the remodeling of tumor immune microenvironment by affecting the interactions between tumor cells and infiltrating immune cells [16–18]. Super-enhancer (SEs) are large spatially clustered transcriptionally active enhancers, typically spanning several kilobases, that can be predicted by strong occupancy signals of specific histone modifications such as H3K27 acetylation (H3K27ac) [19–21]. Enhancer components in SEs are functionally non-redundant which act in a synergistic or additive manner, enabling SEs to drive target genes transcription more robustly than typical enhancers [22–24]. SEs combine with transcription factors to powerfully drive the transcription of genes that control and define cell identity [21]. SEs and master transcription factors that regulate target gene expression are essential for DLBCL progression [25, 26]. However, the precise mechanisms by which SEs regulate the interactions between DBLCL cells and tumor-infiltrating immune cells remain elusive.

This study aimed to investigate the key regulators controlled by SEs in DLBCL cells that regulate the interactions between DLBCL cells and tumor-infiltrating immune cells. We analyzed immune cell clusters with significantly enhanced interactions with B cell cluster in DLBCL. Ligand-receptor interactions of B cell cluster and infiltrating immune cell clusters were identified. Subsequently, we identified SE-controlled ligand-encoding gene and its transcription factor. Finally, we explored the effects of the SE-controlled ligand-encoding gene and its transcription factor in DLBCL cells escape from natural killer (NK) cell killing *in vitro*. This study is expected to provide new therapeutic targets for the treatment of DLBCL.

## Materials and methods

### Single-cell RNA sequencing (scRNA-seq) data analysis

The scRNA-seq gene expression matrix of DLBCL and reactive non-malignant lymph node (rLN) samples were downloaded from the Heidelberg Open Research Data database (heiDATA,^1^) under accession code VRJUNV [14], and the Gene Expression Omnibus database (GEO,^2^) under accession code GSE182434 [15]. Uniform manifold approximation and projection (UMAP) dimensionality reduction analysis of scRNA-seq data was conducted using the “RunUMAP” in R package “Seurat” to generate 2D plots to visualize cell clusters and sub-clusters [27]. Cell clusters and sub-clusters were annotated according to the well-recognized cell-specific markers using CellMarker 2.0 web tool^3^ [28]. The number and strength of interactions among cell clusters or sub-clusters were evaluated using the CellChat v1.6.1.

### Chromatin immunoprecipitation followed by sequencing (ChIP-seq) data analysis

H3K27ac ChIP-seq data of 28 DLBCL cell lines were downloaded from the GSE182214 dataset [25]. Enhancers were defined as the H3K27ac-enriched regions using the “findPeaks” tool in HOMER algorithm. Enhancer constituents clustered within 12.5 kb were stitched together. The “super enhancer” tool in HOMER algorithm was used to rank enhancers according to the H3K27ac signals. Threshold for SE screening was the tangent slope >1 for the rank ordered set. To define the SE-controlled gene, the “annotatePeaks” tool in HOMER algorithm was used to assign enhancers to the nearest genes on the genome. H3K27ac signals at the *CD70* locus were visualized using the UCSC Genome Browser database^4^.

### Survival and immune score analysis

Bulk transcriptomic data and clinical information of 928 DLBCL patients were downloaded from the GSE117556 dataset [29]. Overall survival (OS) and progression-free survival (PFS) of DLBCL patients was assessed by Kaplan-Meier analysis and log-rank test using the X-tile software. Log-rank test P < 0.05 indicated a significant difference. The immune score within the bulk transcriptomic data was calculated using both the CIBERSORT and xCell algorithms. Subsequently, Pearson’s analysis was employed to assess the correlation between MIF, CCL3 and CD70 expression levels and the immune scores.

### Prediction of transcription factor binding sites

Transcription factor binding sites for *CD70* were predicted using the Cistrome Data Browser database^5^ [30, 31].

### Cell culture

The human DLBCL cell line, DOHH2, was purchased from MeisenCTCC (Zhejiang, China) and cultured in RPMI 1640 medium (Gibco, MA, United States) with 10% fetal bovine serum (FBS; Sigma-Aldrich, MO, United States) and 1% penicillin/streptomycin (Invitrogen, CA, United States) at 37°C with 5% CO_2_.

The human NK cell line, NK92, was acquired from American Type Culture Collection (ATCC; VA, United States). NK92 cells were cultured in complete RPMI 1640 medium at 37°C with 5% CO_2_, and activated with 200 U/mL interleukin-2 (IL-2; Sigma-Aldrich, MO, United States).

### JQ1 treatment

DOHH2 cells were seeded into 96-well plates at density of 2 × 10^3^ cells per well and cultured at 37°C for 48 h. The bromodomain and extra-terminal domain (BET) inhibitor JQ1 (Solarbio, Beijing, China) was added into each well to the indicated concentrations (0 or 1 μM) and incubated for 24 h.

### Cell transfection

Small interfering RNAs (siRNAs) targeting CD70 (siCD70) and ZZZ3 (siZZZ3), and negative control siRNA (siNC) were obtained from GenePharma (Shanghai, China). The eukaryotic plasmid pcDNA3.1 for ZZZ3 overexpression (OE-ZZZ3) and the empty pcDNA3.1 plasmid (OE-NC) were synthesized by GenePharma (Shanghai, China). DOHH2 cells were seeded into 6-well plates and cultured until the cell confluency reached approximately 80%. Cell transfection was conducted using Lipofectamine 3000 (Invitrogen, CA, United States) according to the manufacturer’s instructions.

### RNA isolation and quantitative real‐time PCR (qRT-PCR)

Total RNA of DOHH2 cells was isolated using TRIzol reagent (Invitrogen, CA, United States) according to the manufacturer’s instructions. cDNA was generated with 500 ng RNA per reaction using the PrimeScript™ RT Master Mix (Takara, Tokyo, Japan). Quantitative PCR (qPCR) was performed with SYBR Green Master Mix (Takara, Tokyo, Japan). Relative expression levels of CD70 and ZZZ3 were calculated by the 2^−ΔΔCT^ formula with GAPDH as the internal reference. Primers to amplify genes are listed as follows:

*CD70*, forward: 5′-GAC​CCC​AGG​CTA​TAC​TGG​CA-3′; reverse: 5′-CAG​GCT​GAT​GCT​ACG​GGA​G-3’.

*ZZZ3*, forward: 5′-AAA​CGA​GCT​TGT​CGA​TGT​CTT-3′; reverse: 5′-GAC​AGC​CAA​ATA​GCC​TGT​GAT-3′.

*GAPDH*, forward: 5′-GGA​GCG​AGA​TCC​CTC​CAA​AAT-3′; reverse: 5′-GGC​TGT​TGT​CAT​ACT​TCT​CAT​GG-3′.

### ChIP-qPCR

DOHH2 cells were fixed with 1% formaldehyde for 10 min at 25°C and quenched with 0.125 M glycine for 5 min. Cells were lysed with SDS lysis buffer for 10 min at 4°C, and then sonicated using a M220 Focused-ultrasonicator (Covaris, MA, United States) for 10 min in 0.5 min pulse intervals. The ultrasound products were incubated with anti-H3K27ac (ab4729, abcam, United States) or IgG (ab172730, abcam, United States) at 4°C overnight. The immunoprecipitated DNA was purified using the DNA Purification Kit (Beyotime, Shanghai, China), and then subjected to qPCR reactions. Primers used for ChIP-qPCR are listed as follows:

*CD70*-SE1, forward: 5′-CTG​CCA​GTG​GAA​GTG​TTT​GC-3′; reverse: 5′-ACG​TCA​GAA​GTG​CAG​CCT​TT-3′.

*CD70*-SE2, forward: 5′-CAC​GGA​CGT​AAG​CAG​AGA​GG-3′; reverse: 5′-TTT​GCA​GCG​TAG​AGA​GTC​CG-3′.

*CD70*-SE3, forward: 5′-TTC​ACT​GAA​GTG​CCT​CCG​AC-3′; reverse: 5′-TGA​CAG​TTT​GAG​ATG​CCC​CC-3′.

### Cell proliferation assay for DLBCL cells

Cell proliferation of DOHH2 cells was determined using an MTS Assay Kit (abcam, United States). DOHH2 cells were seeded into 96-well plates with 1 × 10^4^ cells per well, and cultured for 0, 1, 2, and 3 days. 10 μL MTS reagent was added into each well at each time point and incubated for 4 h at 37°C. Absorbance at 490 nm (A_490_) was detected using a microplate reader (Bio-Rad, CA, United States).

### Cell proliferation assay for NK cells

NK92 cells were precultured with 200 U/mL IL-2 for activation. DOHH2 cells were treated with 25 μg/mL mitomycin C for 1 h at 37°C to prevent cell proliferation. Mitomycin C pretreated DOHH2 cells were co-cultured with activated NK92 cells at a ratio of 1:1, 2:1 and 5:1 in RPMI 1640 medium for 48 h. Proliferation of NK92 cells was determined using an MTS Assay Kit (abcam, United States) according to the manufacturer’s instructions. A_490_ was detected using a microplate reader (Bio-Rad, CA, United States).

### Lactate dehydrogenase (LDH) release assay

DOHH2 cells were co-cultured with IL-2 activated NK92 cells as indicated ratios for 48 h. The co-culture systems were treated with or without anti-PD-L1 (ab205921, abcam, United States) or IgG (ab172730, abcam, United States). LDH release were measured using the Cytotoxicity LDH Assay Kit-WST (Dojindo, Kyushu, Japan) according to the manufacturer’s instructions. Absorbance at 490 nm was detected using a microplate reader (Bio-Rad, CA, United States).

### Statistical analysis

Statistical data were analyzed using R software (version 4.1.2) and GraphPad Prism (version 9.0), and presented as mean ± standard deviation (SD). Differences among multiple groups were analyzed by one-way analysis of variance (ANOVA) followed by Tukey’s *post hoc* test. Differences between two groups were analyzed by Student’s *t*-test. P < 0.05 indicated a statistical significance.

## Results

### Five cell clusters were identified in DLBCL and rLN

**FIGURE 1:**
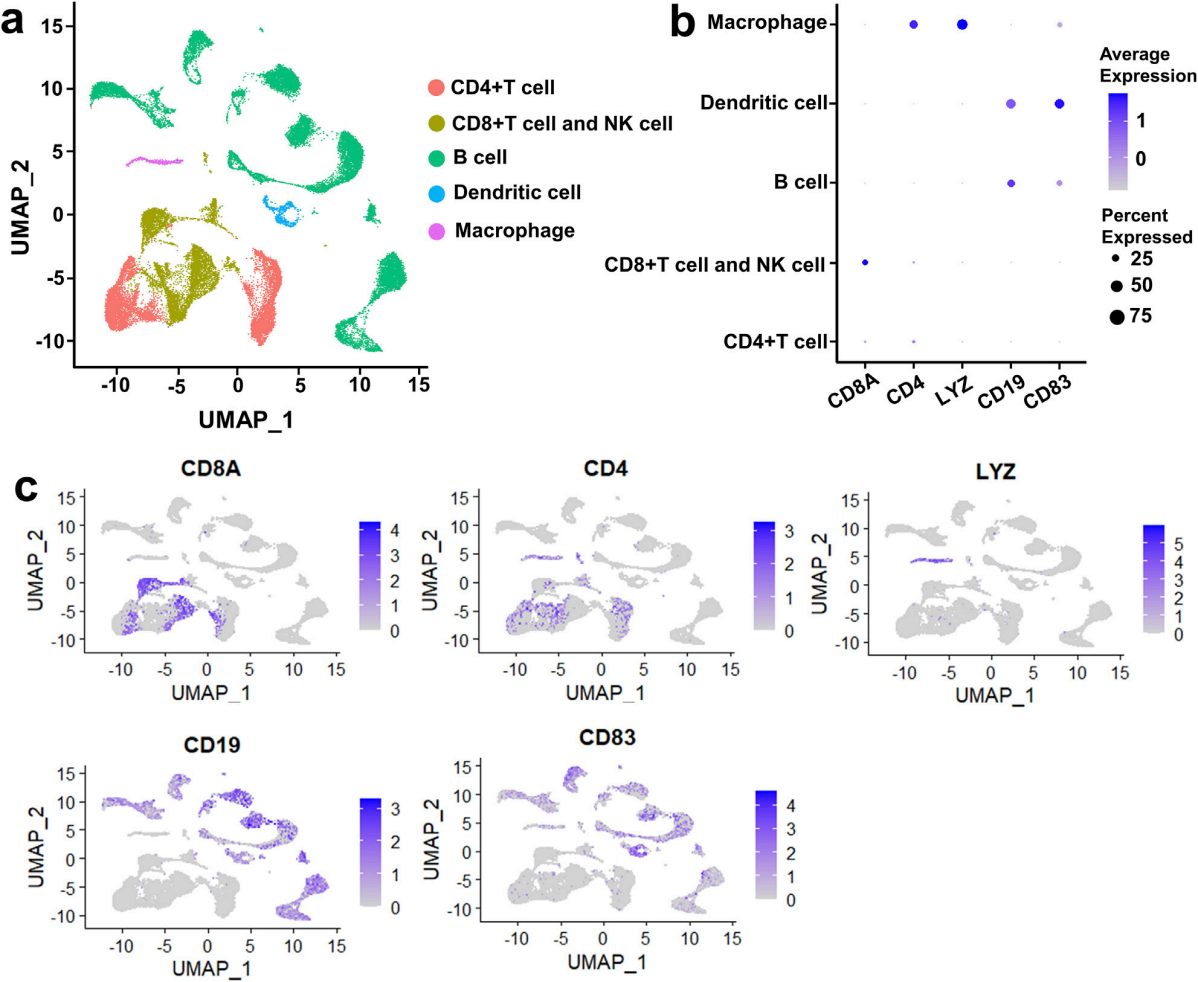
Five cell clusters were identified in DLBCL and rLN based on the VRJUNV and GSE182434 datasets. **(A)** dimensionality reduction was performed by UMAP. **(B)** Dot plots depicting the expression levels of cell-specific markers in each cell cluster, as well as the expression percentage of the markers. **(C)** Feature plots showed the expression of cell-specific markers in global cell clusters.

Immune microenvironment plays a crucial role in the tumorigenesis of DLBCL. To describe the heterogeneity of the immune microenvironment in DLBCL, we performed UMAP dimensionality reduction on DLBCL and rLN samples according to the VRJUNV and GSE182434 datasets. Dimensionality reduction by UMAP resulted in five cell clusters, including CD4‐expressing (CD4^+^) T cell cluster, CD8‐expressing (CD8^+^) T cell and NK cell cluster, B cell cluster, dendritic cell cluster, and macrophage cluster (Figure 1A). The five cell clusters were identified by unique cell-specific marker genes expression as follow: CD8A for CD8^+^ T cells and NK cells, CD4 for CD4^+^ T cells, LYZ for macrophages, CD19 for B cells, and CD83 for dendritic cells (Figure 1B). Subsequently, these clusters were reclassified into global cellular compartments based on the expression of cell-specific markers (Figure 1C). Taken together, five cell clusters, including CD4^+^ T cell cluster, CD8^+^ T cell and NK cell cluster, B cell cluster, dendritic cell cluster, and macrophage cluster, were identified in DLBCL and rLN.

### The enhanced interaction of B cell cluster to CD8^+^ T cell and NK cell cluster was most prominent in DLBCL compared with rLN

**FIGURE 2:**
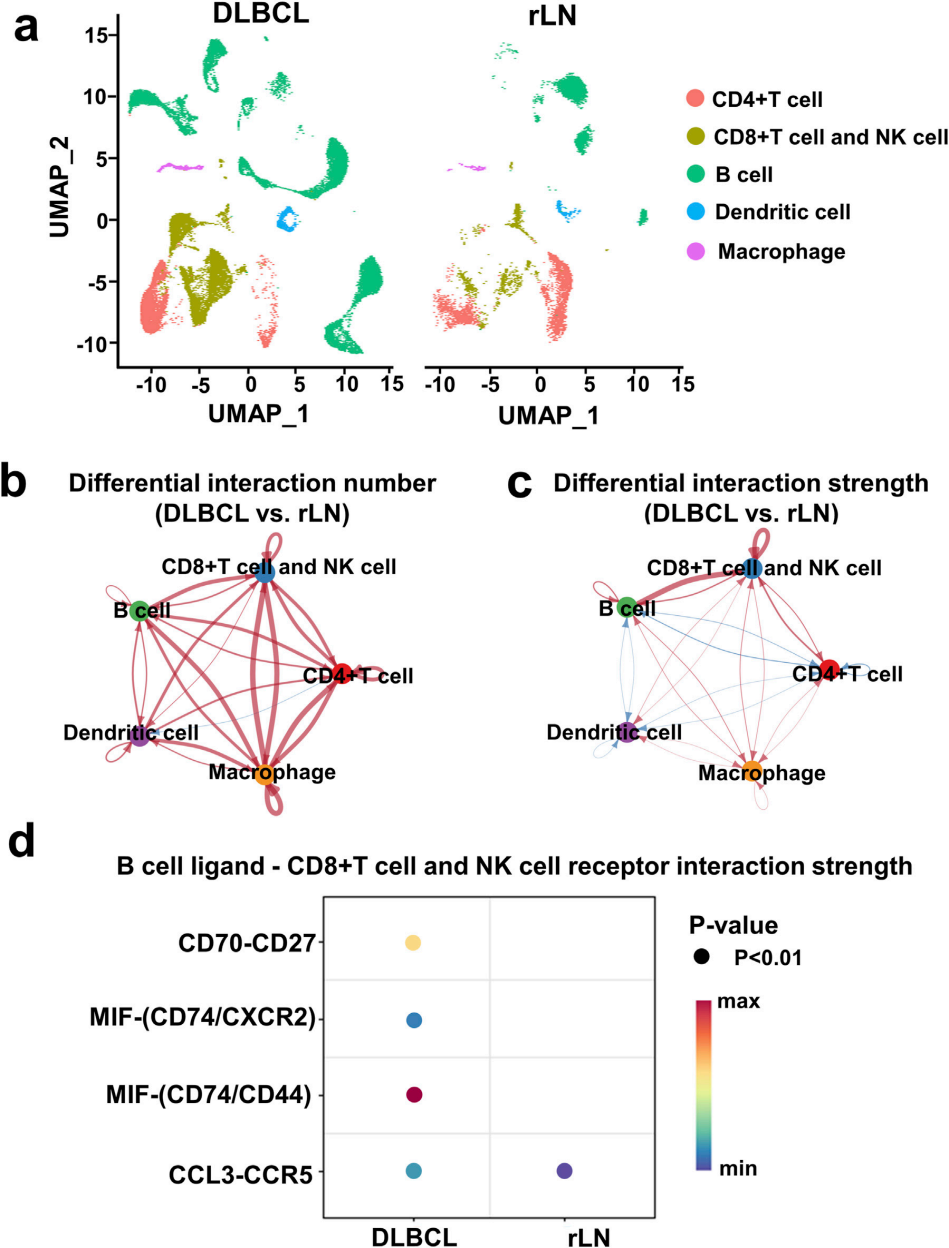
Interaction analysis of five cell clusters in DLBCL compared with rLN based on the VRJUNV and GSE182434 datasets. **(A)** UMAP plots of five cell clusters in DLBCL and rLN were analyzed respectively. **(B, C)** Networks of differential interaction numbers **(B)** and strengths **(C)** in DLBCL compared with rLN. Red lines represented upregulation of interaction number or strength in DLBCL compared with rLN, while blue lines represented downregulation. The thicker the line, the greater difference in the interaction number or strength. **(D)** Dot plots of B cell ligands and CD8^+^ T cell and NK cell receptors interactions that were significantly different between DLBCL and rLN.

To investigate DLBCL-specific immune cell interactions, we described immune cell clusters in DLBCL and rLN respectively (Figure 2A). Subsequently, we analyzed the differences in the number and strength of cell cluster interactions between DLBCL and rLN. The interaction numbers of B cell cluster to the other four cell clusters (CD4^+^ T cell cluster, CD8^+^ T cell and NK cell cluster, dendritic cell cluster, and macrophage cluster) were increased in DLBCL compared with rLN (Figure 2B). The interaction strengths of B cell cluster to CD4^+^ T cell cluster and dendritic cell cluster were attenuated in DLBCL compared with rLN (Figure 2C). However, the interaction strengths of B cell cluster to macrophage cluster and CD8^+^ T cell and NK cell cluster were enhanced in DLBCL compared with rLN (Figure 2C). Especially, the interaction strength of B cell cluster to CD8^+^ T cell and NK cell cluster was most dramatically enhanced in DLBCL compared with rLN (Figure 2C). Furthermore, we analyzed the differential interactions between B cell ligands and CD8^+^ T cell and NK cell receptors in DLBCL and rLN. A total of four pairs of ligand-receptor interactions (including CD70-CD27, MIF-CD74/CXCR2 complex, MIF-CD74/CD44 complex, and CCL3-CCR5) were significantly upregulated in DLBCL compared with rLN (Figure 2D). Collectively, these findings suggested that the enhanced interaction of B cell cluster to CD8^+^ T cell and NK cell cluster was most prominent in DLBCL compared with rLN.

### Strong interaction was found between B cell cluster and NK cell sub-cluster in DLBCL

**FIGURE 3:**
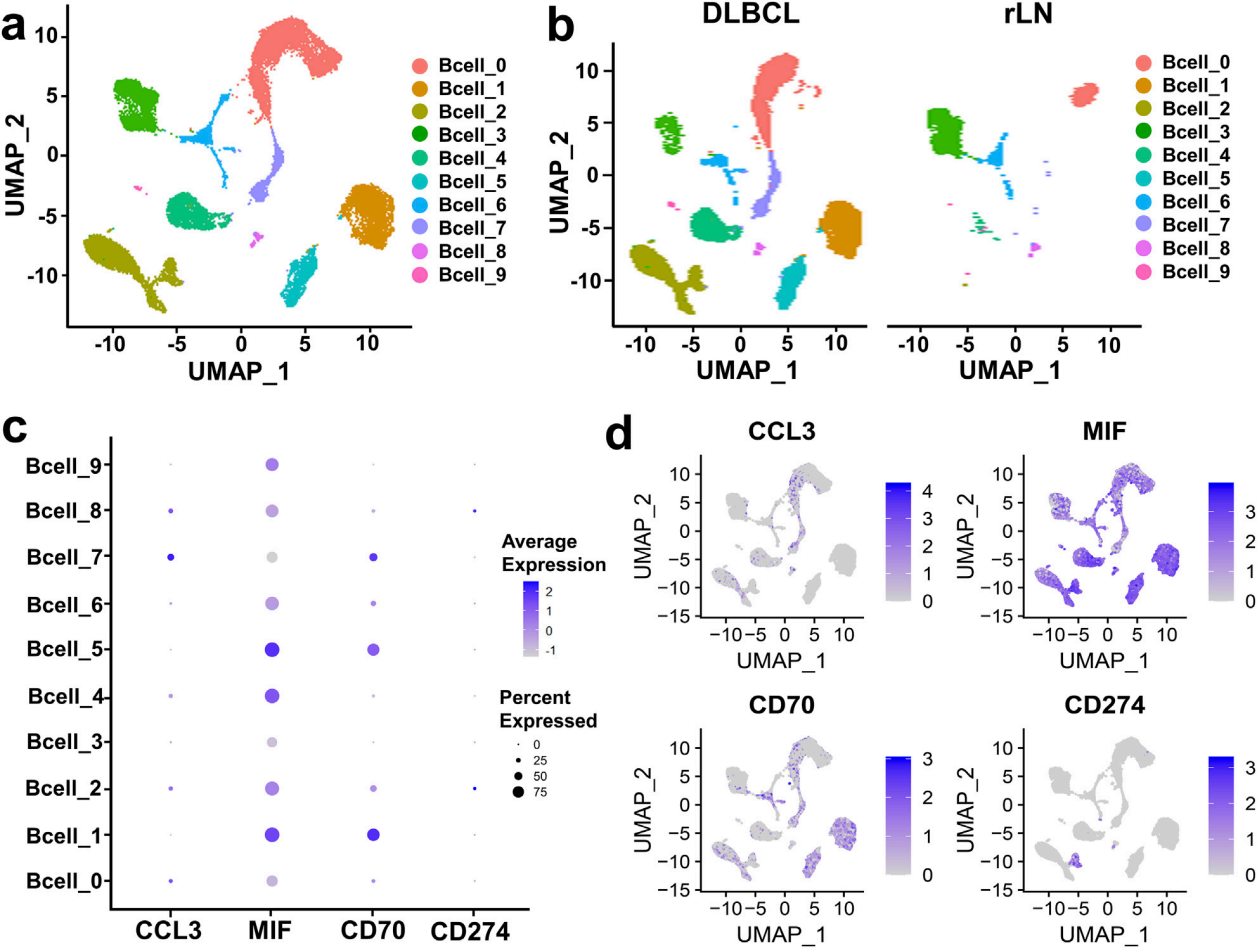
Expression and distribution analysis of CCL3, MIF, CD70, and CD274 in the ten B cell sub-clusters based on the VRJUNV and GSE182434 datasets. **(A)** UMAP plots of the ten sub-clusters of B cell cluster in DLBCL and rLN. **(B)** UMAP dimensionality reduction plots for DLBCL and rLN, respectively. **(C)** Dot plots of the expression levels and percentages of CCL3, MIF, CD70, and CD274 in the ten B cell sub-clusters. **(D)** Feature plots of the distribution of CCL3, MIF, CD70, and CD274 expression in B cell cluster.

To further investigate the interaction of B cell cluster with CD8^+^ T cell and NK cell cluster, we classified B cell cluster into ten sub-clusters based on the VRJUNV and GSE182434 datasets (Figure 3A). Dimensionality reduction was performed on B cell sub-clusters of DLBCL and rLN, respectively (Figure 3B). Then, we analyzed the expression of ligand-encoding genes in the upregulated ligand-receptor interaction pairs. CD70 was mainly expressed in Bcell_1, Bcell_2, Bcell_5, Bcell_6 and Bcell_7 sub-clusters; CCL3 was mainly expressed in Bcell_0, Bcell_2, Bcell_4, Bcell_7 and Bcell_8 sub-clusters; MIF was expressed in all of the ten sub-clusters (Figures 3C, D). Additionally, we analyzed the expression of *CD274* (PD-L1 encoding gene) in each B cell sub-cluster. The results showed that *CD274* was mainly expressed in Bcell_2 and Bcell_8 sub-clusters (Figures 3C, D).

**FIGURE 4:**
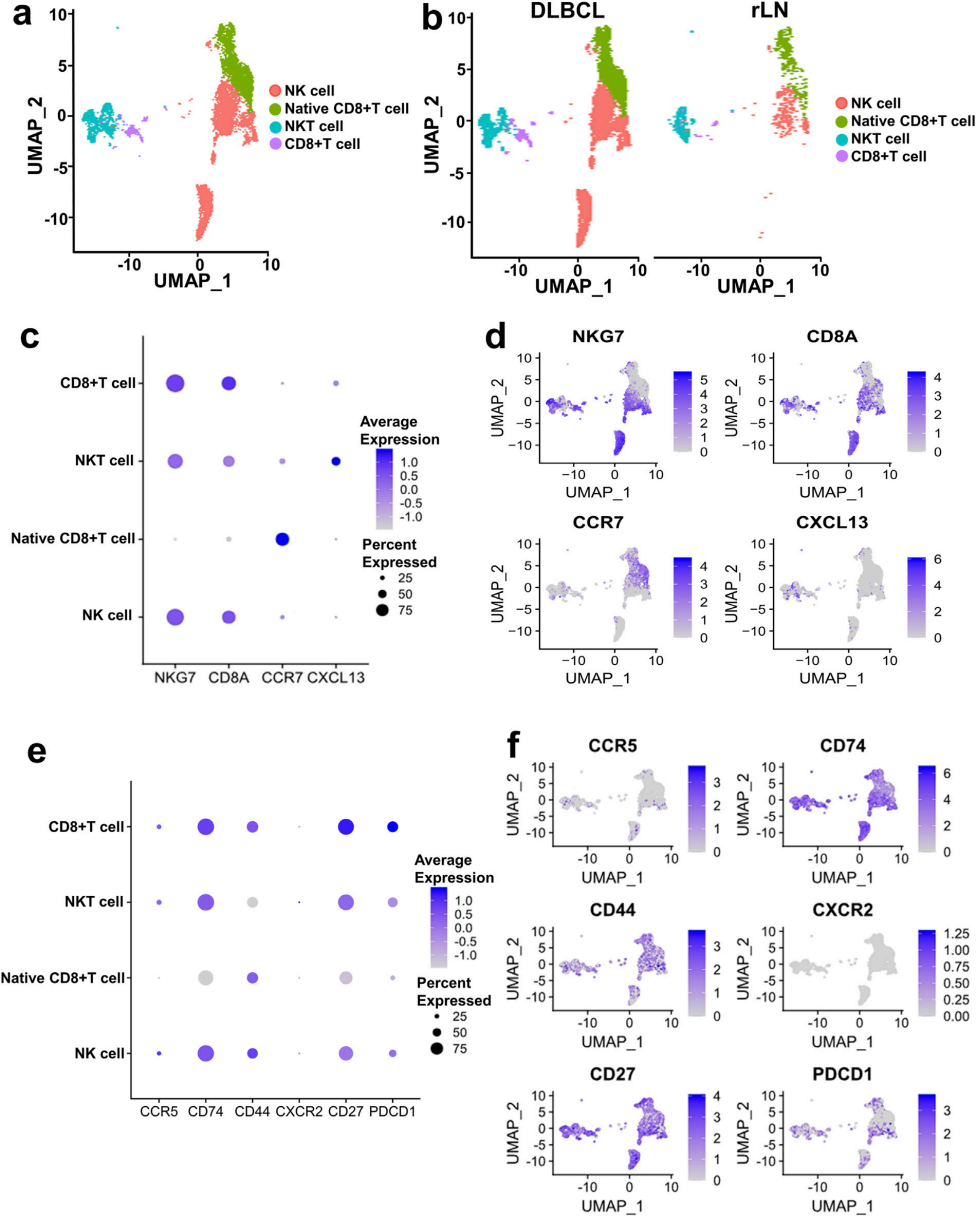
Expression and distribution analysis of CCR5, CD74, CD44, CXCR2, CD27, and PDCD1 in the four sub-clusters of CD8^+^ T cell and NK cell cluster based on the VRJUNV and GSE182434 datasets. **(A)** UMAP plots of the distribution of the four sub-clusters of CD8^+^ T cell and NK cell cluster (NK cell sub-cluster, native CD8^+^ T cell sub-cluster, NKT cell sub-cluster, and CD8^+^ T cell sub-cluster) in DLBCL and rLN. **(B)** UMAP plots of NK cell, native CD8^+^ T cell, NKT cell, and CD8^+^ T cell sub-clusters distribution in DLBCL and rLN, respectively. **(C)** Dot plots of specific marker genes expression in the four sub-clusters of CD8^+^ T cell and NK cell cluster together with the expression percentage of the marker genes. **(D)** Feature plots of specific marker genes expression in the four sub-clusters of CD8^+^ T cell and NK cell cluster. **(E)** Expression levels and percentages of CCR5, CD74, CD44, CXCR2, CD27, and PDCD1 in the four sub-clusters. **(F)** Distribution of CCR5, CD74, CD44, CXCR2, CD27, and PDCD1 expression in the CD8^+^ T cell and NK cell cluster.

CD8^+^ T cell and NK cell cluster of DLBCL and rLN was classified into four sub-clusters including NK cell sub-cluster, native CD8^+^ T cell sub-cluster, NKT cell sub-cluster, and CD8^+^ T cell sub-cluster (Figures 4A, B). We calculated the expression distribution of specific marker genes of the four sub-clusters: NKG7 for NK cells, CD8A for CD8^+^ T cells, CCR7 for native CD8^+^ T cells, and CXCL13 for NKT cells (Figures 4C, D). These results indicated that we obtained reliable annotations of sub-clusters of CD8^+^ T cell and NK cell cluster. Then, we analyzed the expression of receptor-encoding genes in the upregulated ligand-receptor interaction pairs. CD27, CD74 and CCR5 were mainly expressed in NK cell sub-cluster, NKT cell sub-cluster, and CD8^+^ T cell sub-cluster (Figures 4E, F). CD44 was mainly expressed in NK cell sub-cluster, native CD8^+^ T cell sub-cluster, and CD8^+^ T cell sub-cluster (Figures 4E, F). CXCR2 was mainly expressed in NKT cell sub-cluster (Figures 4E, F). PD1-encoding gene, *PDCD1*, was mainly expressed in NK cell sub-cluster, NKT cell sub-cluster, and CD8^+^ T cell sub-cluster (Figures 4E, F).

**FIGURE 5:**
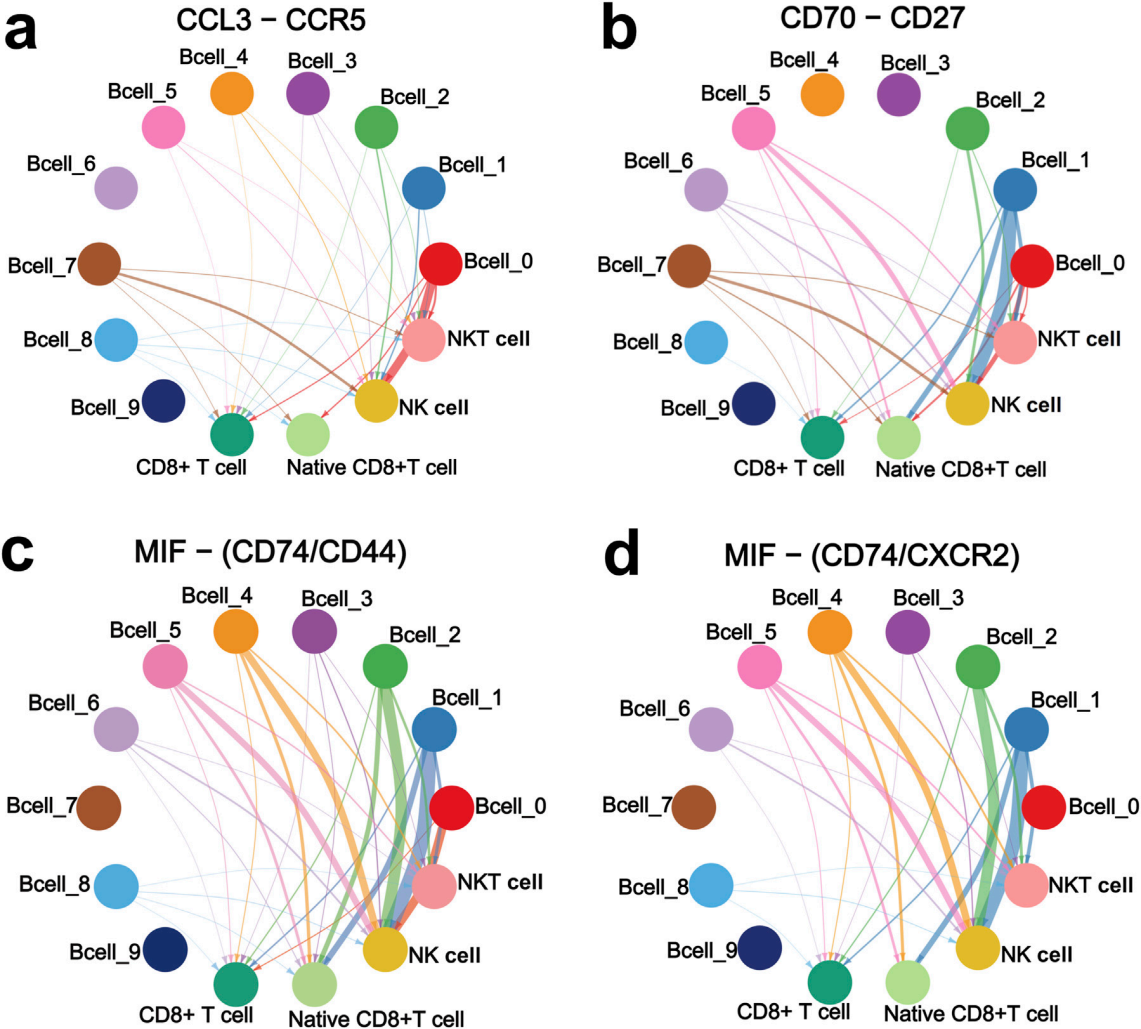
CCL3-CCR5 **(A)**, CD70-CD27 **(B)**, MIF-CD74/CD44 complex **(C)**, and MIF-CD74/CXCR2 complex **(D)** interaction networks of the ten B cell sub-clusters with NK cell, native CD8^+^ T cell, NKT cell, and CD8^+^ T cell sub-clusters. Interaction networks were constructed based on the VRJUNV and GSE182434 datasets. The thicker the line, the stronger the interaction.

Furthermore, we analyzed the interactions of the four upregulated interacting ligand-receptor pairs between the ten B cell sub-clusters and the four CD8^+^ T cell and NK cell sub-clusters. CCL3 and CCR5 showed strong interactions in Bcell_0 and Bcell_7 sub-clusters to NK cell sub-cluster (Figure 5A). CD70 and CD27 showed strong interactions in Bcell_1, Bcell_2, Bcell_5 and Bcell_7 sub-clusters to NK cell sub-cluster (Figure 5B). Interactions of MIF to CD74/CD44 receptor complex were strong in Bcell_0, Bcell_1, Bcell_2, Bcell_4 and Bcell_5 sub-clusters to NK cell sub-cluster (Figure 5C). Interactions of MIF to CD74/CXCR2 receptor complex were strong in Bcell_1, Bcell_2, Bcell_4 and Bcell_5 sub-clusters to NK cell sub-cluster (Figure 5D). Importantly, CCL3-CCR5, CD70-CD27, MIF-CD74/CD44 complex, and MIF-CD74/CXCR2 complex interacted more strongly between B cell cluster and NK cell sub-cluster than interactions between B cell cluster and native CD8^+^ T cell sub-cluster, NKT cell sub-cluster, or CD8^+^ T cell sub-cluster (Figure 5).

To further validate the impact of MIF, CCL3 and CD70 expression on the prognosis of DLBCL patients, we analyzed bulk transcriptomic data from the GSE117556 dataset. High MIF expression indicated lower OS, but showed no significant difference in PFS (Supplementary Figures S1A, B). Low CCL3 expression was associated with worse PFS, but OS did not show any statistical difference (Supplementary Figures S1C, D). Patients with high CD70 expression exhibited significantly worse OS and PFS compared to those with low CD70 expression (Supplementary Figures S1E, F). Additionally, immune scores were calculated based on bulk transcriptomic data using CIBERSORT and xCell algorithms. Pearson’s analysis was performed to correlate the immune scores with MIF, CCL3 and CD70 expression levels. Integrating the results of the two algorithms, we found that MIF had the strongest correlation with B cell immune scores, while CCL3 correlated most strongly with CD8^+^ T cells and macrophages (Supplementary Figure S2). CD70 showed the strongest correlation with CD8^+^ T cells, but the correlation coefficient was relatively low (R < 0.3) (Supplementary Figure S2). Unfortunately, we did not find a significant correlation between MIF, CCL3 and CD70 with NK cell immune scores (Supplementary Figure S2).

Taken together, the interaction between B cell cluster and NK cell sub-cluster was the strongest in DLBCL, and this interaction may have potential clinical significance. Therefore, we focused on this interaction for further investigation.

### CD70 was identified as a SE-controlled gene in DOHH2, HBL1, and NU-DHL1 cells

**FIGURE 6:**
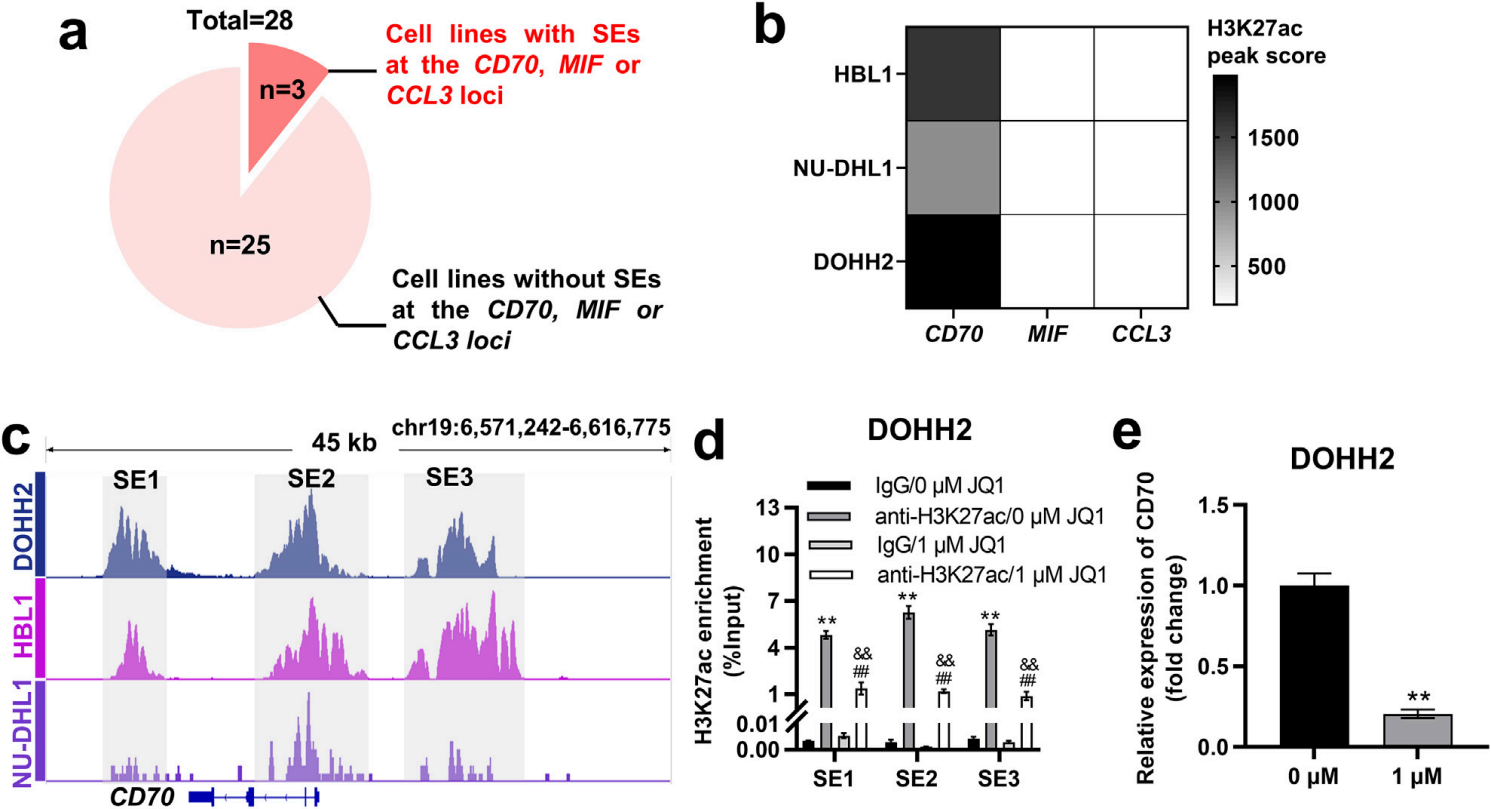
*CD70* was identified as a SE-controlled gene in DOHH2, HBL1 and NU-DHL1 cells. **(A)** Screening of DLBCL cell lines with SE at the *CD70*, *MIF* or *CCL3* locus based on the GSE182214 dataset. **(B)** Heatmap of H3K27ac peak score for SE at the *CD70*, *MIF* or *CCL3* locus in HBL1, NU-DHL1, and DOHH2 cells based on the GSE182214 dataset. **(C)** Identification of three SE regions (SE1, SE2, and SE3) at the *CD70* locus in HBL1, NU-DHL1, and DOHH2 cells based on the GSE182214 dataset. **(D)** ChIP-qPCR was used to analysis the H3K27ac enrichment in three SE regions, and the effect of JQ1 treatment on H3K27ac enrichment in SE regions in DOHH2 cells. **P < 0.01, anti-H3K27ac/0 μM JQ1 group vs. IgG/0 μM JQ1 group. ##P < 0.01, anti-H3K27ac/1 μM JQ1 group vs. IgG/1 μM JQ1 group. &&P < 0.01, anti-H3K27ac/1 μM JQ1 group vs. anti-H3K27ac/0 μM JQ1 group. **(E)** qRT-PCR was used to detect the relative expression of CD70 in DOHH2 cells treated with 0 or 1 μM JQ1. **P < 0.01, 1 μM JQ1 group vs. 0 μM JQ1 group.

SEs are key factors driving oncogene expression in tumor cells [22–24]. We wondered whether the expression of CD70, MIF and CCL3 in DLBCL cells is regulated by SEs. H3K27ac signals at the *CCL3*, *CD70* and *MIF* loci in 28 DLBCL cell lines were analyzed based on ChIP-seq data from the GSE182214 dataset. The results showed that SE was present at the *CCL3*, *CD70* or *MIF* locus in 3 DLBCL cell lines, whereas it was absent in the other 25 cell lines (Figure 6A). HBL1, NU-DHL1 and DOHH2 cells were found to exhibit SE only at the *CD70* locus, but not at the *MIF* and *CCL3* loci (Figure 6B). The 3 cell lines were ranked in descending order according to the H3K27ac peak score for SE at the *CD70* locus as follows: DOHH2, HBL1, and NU-DHL1 cells (Figure 6B). We identified SE at the *CD70* locus in DOHH2, HBL1 and NU-DHL1 cells based the GSE182214 dataset. SE of *CD70* was divided into three regions (SE1, SE2, and SE3) based on the enrichment of H3K27ac signals (Figure 6C). Since DOHH2 cells had the highest H3K27ac peak score for SE of *CD70*, we validated the SE regions at the *CD70* locus in DOHH2 cells using ChIP-qPCR. H3K27ac in SE1, SE2 and SE3 regions was significantly enriched in DOHH2 cells (Figure 6D). JQ1 treatment significantly inhibited H3K27ac enrichment in the three SE regions (Figure 6D), and significantly downregulated *CD70* transcription in DOHH2 cells (Figure 6E). Collectively, CD70 was identified as a SE-controlled gene in DLBCL cells.

### ZZZ3 interacted with the SE of CD70 to drive CD70 expression

**FIGURE 7:**
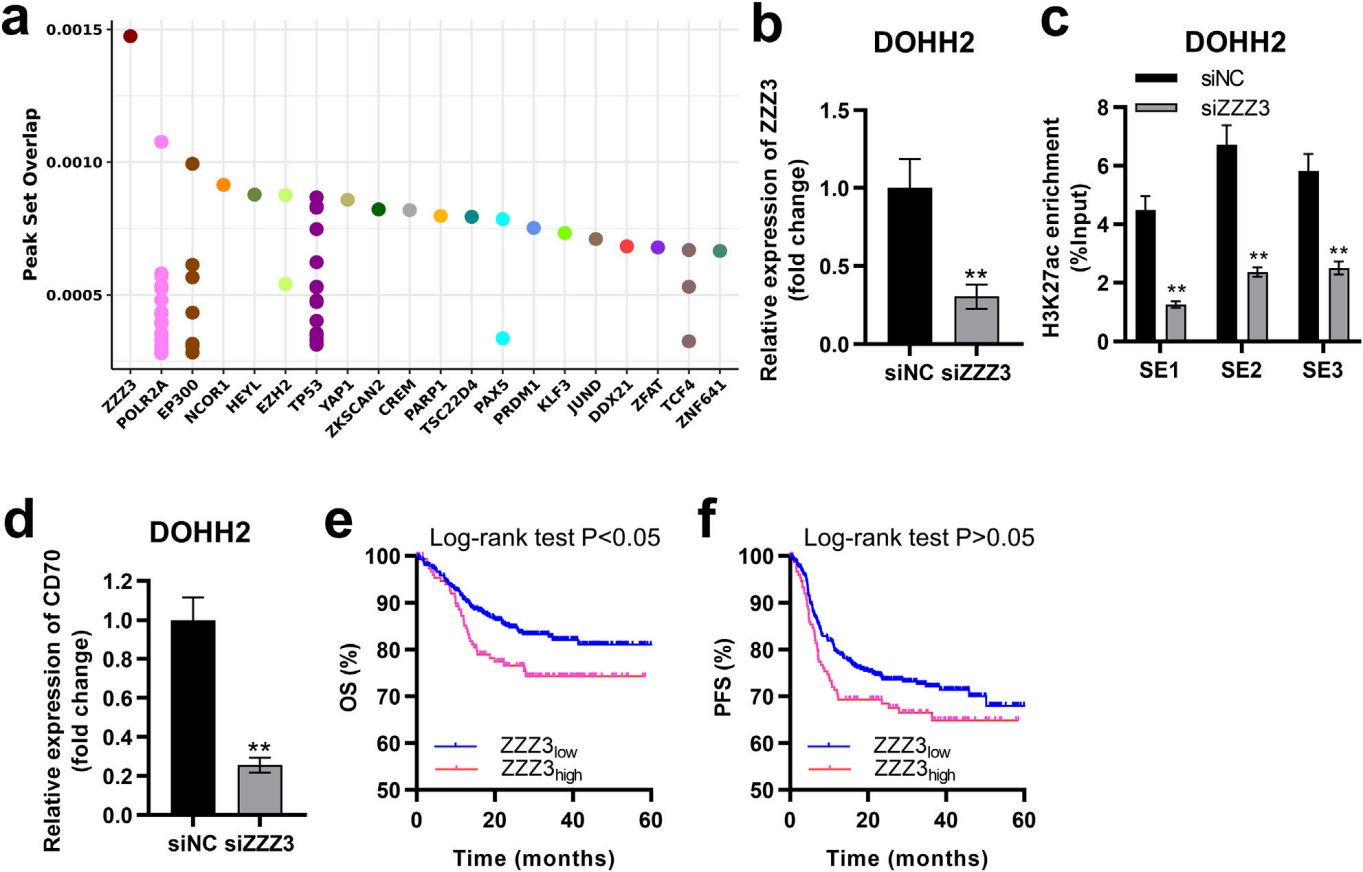
ZZZ3 interacted with the SE of *CD70* to drive CD70 expression. **(A)** Dot plots of the predicted transcription factors of *CD70*. **(B)** qRT-PCR was performed to measure the expression of ZZZ3 in DOHH2 cells transfected with siNC or siZZZ3. **P < 0.01, siZZZ3 group vs. siNC group. **(C)** ChIP-qPCR was used to detect the H3K27ac enrichment in three SE regions at the *CD70* locus in DOHH2 cells transfected with siNC or siZZZ3. **P < 0.01, siZZZ3 group vs. siNC group. **(D)** qRT-PCR was performed to measure the expression of CD70 in DOHH2 cells transfected with siNC or siZZZ3. **P < 0.01, siZZZ3 group vs. siNC group. **(E,F)** Kaplan–Meier overall survival (OS) and progression-free survival (PFS) curves of DLBCL patients with high- and low-ZZZ3 expression were plotted based on the GSE117556 dataset.

SEs driving transcription of target genes must be recognized and bound by transcription factors [21]. Potential transcription factors regulating *CD70* were ranked in descending order according to the peak set overlap score, with ZZZ3 ranking first (Figure 7A). We successfully established ZZZ3-silenced cells by transfecting siZZZ3 into DOHH2 cells (Figure 7B). Silencing ZZZ3 significantly inhibited H3K27ac enrichment in SE1, SE2 and SE3 regions of the *CD70* locus in DOHH2 cells (Figure 7C). Moreover, silencing ZZZ3 significantly suppressed *CD70* transcription in DOHH2 cells (Figure 7D). DLBCL patients with high-ZZZ3 expression have significantly worse OS than patients with low-ZZZ3 expression (Figure 7E). High-ZZZ3 expression is associated with a poor PFS of DLBCL, although not significantly (descriptive Log-rank P > 0.05; Figure 7F). Taken together, ZZZ3 bound to SE of *CD70* to drive *CD70* transcription.

### The ZZZ3/CD70 axis in DLBCL cells promoted their escape from NK cell killing

**FIGURE 8:**
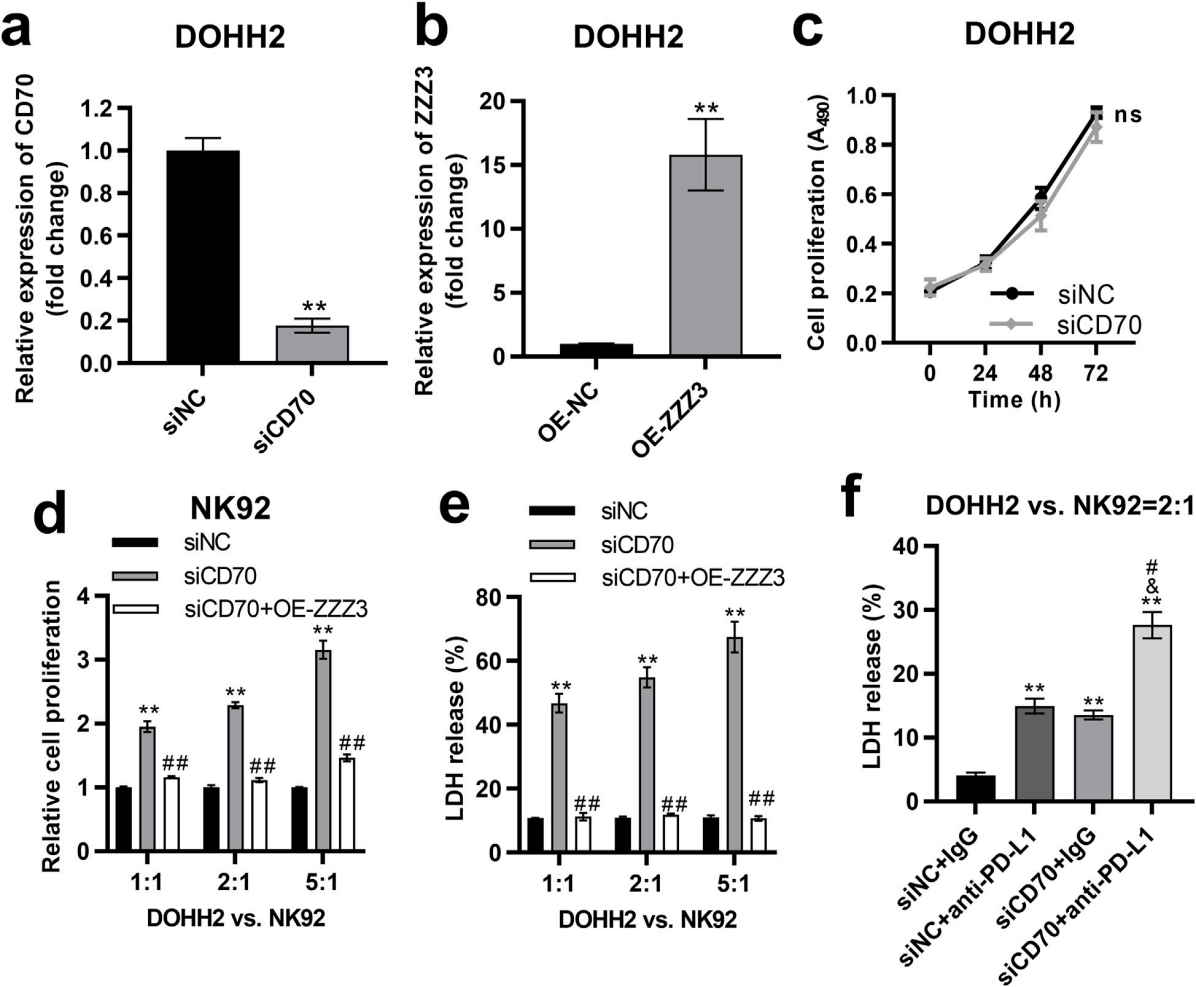
The ZZZ3/CD70 axis in DLBCL cells promoted their escape from NK cell killing. **(A)** qRT-PCR was performed to measure the expression of CD70 in DOHH2 cells transfected with siNC or siCD70. **P < 0.01, siCD70 group vs. siNC group. **(B)** qRT-PCR was used to measure ZZZ3 expression in DOHH2 cells transfected with OE-NC or OE-ZZZ3. **P < 0.01, OE-ZZZ3 group vs. OE-NC group. **(C)** MTS assay was used to determine the proliferation of DOHH2 cells transfected with siNC or siCD70. Ns. non-significantly. **(D)** MTS assay was used to detect the proliferation of NK92 cells co-cultured with mitomycin C pretreated DOHH2 cells. DOHH2 cells transfected with siNC, siCD70, or co-transfected siCD70 and OE-ZZZ3 (mitomycin C pretreated) and NK92 cells (IL-2 activated) were co-cultured at ratios of 1:1, 2:1 and 5:1 for 48 h **P < 0.01, siCD70 group vs siNC group. ##P < 0.01, siCD70 + OE-ZZZ3 group vs. siCD70 group. **(E)** LDH release from DOHH2 cells co-cultured with IL-2 activated NK92 cells. DOHH2 cells transfected with siNC, siCD70, or co-transfected with siCD70 and OE-ZZZ3 (without mitomycin C pretreatment) were co-cultured with IL-2 activated NK92 cells at ratios of 1:1, 2:1, 5:1. **P < 0.01, siCD70 group vs. siNC group. ##P < 0.01, siCD70 + OE-ZZZ3 group vs. siCD70 group. **(F)** LDH release from DOHH2 cells co-cultured with IL-2 activated NK92 cells and treated with anti-PD-L1 or IgG. DOHH2 cells transfected with siNC or siCD70 (without mitomycin C pretreatment) were co-cultured with IL-activated NK92 cells at a ratio of 2:1. **P < 0.01, vs. siNC + IgG group. &P < 0.05, vs. siNC+anti-PD-L1 group. #P < 0.05, vs. siCD70 + IgG group.

To investigate the impact of the ZZZ3/CD70 axis on DLBCL cell proliferation, we established CD70-silenced and ZZZ3-overexpressing cells by transfecting siCD70 or ZZZ3 overexpression plasmids (OE-ZZZ3) into DOHH2 cells, respectively. Transfection of siCD70 significantly inhibited CD70 expression in DOHH2 cells (Figure 8A). Compared with the DOHH2 cells transfected with the empty pcDNA3.1 plasmids (OE-NC), ZZZ3 expression was significantly upregulated in DOHH2 cells transfected with OE-ZZZ3 (Figure 8B). The effect of CD70 silencing on the proliferation of DOHH2 cells was determined by MTS assay. Silencing CD70 had no significant effect on the proliferation of DOHH2 cells (Figure 8C).

To investigate the impact of the ZZZ3/CD70 axis in DLBCL cells on their resistance to NK cell-mediated killing, we pretreated DOHH2 cells transfected with siNC, siCD70, or co-transfected with siCD70 and OE-ZZZ3 with mitomycin C to block cell proliferation. The mitomycin C pretreated DOHH2 cells were co-cultured with IL-2 activated NK92 cells at ratios of 1:1, 2:1 and 5:1. The proliferation of NK92 cells co-cultured with DOHH2 cells was detected by MTS assay. Co-culture of CD70-silenced DOHH2 cells with NK92 cells significantly promoted the proliferation of NK92 cells compared with NK92 cells co-cultured with DOHH2 cells transfected with siNC (Figure 8D). However, overexpression of ZZZ3 in DOHH2 cells significantly attenuated the promotion of NK92 cell proliferation by CD70 silence of DOHH2 cells in the co-culture system (Figure 8D).

Then, we evaluated the effect of ZZZ3/CD70 axis on LDH release from DOHH2 cells co-cultured with NK92 cells. DOHH2 cells (without mitomycin C pretreatment) were co-cultured with IL-2 activated NK92 cells at ratios of 1:1, 2:1 and 5:1. Silencing CD70 in DOHH2 cells significantly elevated LDH release from DOHH2 cells co-cultured with NK92 cells, which was offset by overexpression of ZZZ3 in DOHH2 cells (Figure 8E). Since interaction between PD1 and PD-L1 contributes to tumor immune evasion [11], we assessed the effect of silencing CD70 in DOHH2 cells combined with anti-PD-L1 treatment on LDH release from DOHH2 cells co-cultured with NK92 cells. DOHH2 cells (without mitomycin C pretreatment) and NK92 cells (IL-2 activated) were co-cultured at a ratio of 2:1. Anti-PD-L1 treatment or silencing CD70 in DOHH2 cells significantly promoted LDH release from DOHH2 cells co-cultured with NK92 cells (Figure 8F). Silencing CD70 in DOHH2 cells combined with anti-PD-L1 treatment significantly promoted LDH release from DOHH2 cells co-cultured with NK92 cells more strongly than anti-PD-L1 treatment alone (Figure 8F).

Taken together, the ZZZ3/CD70 axis in DLBCL cells promoted their escape from NK cell killing.

## Discussion

Interactions between DLBCL cells and tumor-infiltrating immune cells are closely related to the development of DLBCL [16–18]. SEs are key regulators in promoting the malignant phenotype of tumor cells [25, 26], but the roles of SEs in modulating the interactions between tumor cells and infiltrating immune cells remain unknown. In the present study, we found that the enhanced interaction between B cell cluster and CD8^+^ T cell and NK cell cluster in DLBCL compared to rLN. Specific interactions included the CD70-CD27, which contributes to the pathophysiology of autoimmunity [32]; the MIF-CD74/CXCR2 complex, regulating immune cell migration and inflammation [33]; the MIF-CD74/CD44 complex, mediating multiple biological processes, including cell proliferation and the inflammatory response [33, 34]; and the CCL3-CCR5, mediating immune cell recruitment [35]. Notably, a strong interaction between the B cell cluster and NK cell sub-cluster was identified. *CD70* was screened as a SE-controlled gene which was regulated by transcription factor ZZZ3 in DLBCL cells. These results highlighting the role of the CD70-CD27 interaction in DLBCL cell evasion from NK cell killing. Finally, effect of the ZZZ3/CD70 axis on the evasion of DLBCL cells from NK cell killing were examined *in vitro*.

DLBCL is a disease with complex pathogenesis, which is reflected not only in the genetic and epigenetic alterations of B lymphocytes, but also in the complicated crosstalk between tumor cells and tumor-infiltrating immune cells [36].In this study, we characterized the immune cell profile of DLBCL and identified five cell clusters (CD4^+^ T cell cluster, CD8^+^ T cell and NK cell cluster, B cell cluster, dendritic cell cluster, and macrophage cluster). CD4^+^ T cells have cytotoxic or immunoregulatory functions [37, 38]. A low proportion of CD4^+^ T cells in the TME is associated with a poor prognosis of primary central nervous system DLBCL [39]. CD8^+^ T cells exert specific cytotoxic effects by secreting cytokines, releasing perforin and granzyme to kill tumor cells [40]. NK cells express cell surface receptors with stimulatory or inhibitory functions, or secrete cytokines and chemokines to exert cytolytic activity against target cells [41–43]. Dendritic cells exhibit strong antigen-presenting capacity, and stimulate T cells activation to trigger immune responses [44]. Macrophages are key regulators in mediating tumor immune evasion [45]. However, the interactions between DLBCL cells and tumor-infiltrating immune cells remain largely unknown.

Recent studies have successfully created single-cell transcriptome atlases for DLBCL. These atlases reveal phenotypic diversity within DLBCL cases and interactions between tumor cells and the microenvironment. Steen et al. associate CXCR5+ CD8 T cells with the effectiveness of bortezomib when added to standard therapy [15]. Roider et al. propose that malignant B cells can receive both costimulatory and coinhibitory signals from all major T-cell subsets via CD80/CD86-CD28 and CD80/CD86-CTLA4 interactions [14]. In this study, we found that the enhanced interaction of B cell cluster to CD8^+^ T cell and NK cell cluster was most pronounced in DLBCL compared with rLN. Furthermore, we found that NK cell sub-cluster interacted most strongly with B cell cluster. NK cells are innate lymphocytes, which are considered to be the first line of defense for host immune detection and play important roles in the progression of malignant tumors [46–48]. The number and activity of tumor-infiltrating NK cells have significant impacts on the prognosis of various cancers [48–50]. Patients with NK cell dysfunction have higher cancer incidence rates [51, 52]. Interactions between tumor cells and NK cells regulate the phenotype of NK cells, thereby affecting NK cells viability or function [51–55].

NK cells have received increasing attention for their potential in immunotherapy. However, studies on NK cells in DLBCL remain scarce. Frank Vari et al found that NK cell-mediated immune evasion is achieved by the interaction of PD1 and PD-L1 between NK cells and DLBCL cells [11]. However, the direct crosstalk and regulatory mechanisms of DLBCL cell-NK cell interaction remain largely enigmatic. Herein, we found that the interactions of CCL3-CCR5, CD70-CD27, MIF-CD74/CD44 complex, and MIF-CD74/CXCR2 complex between B cell cluster and CD8^+^ T cells and NK cell cluster were significantly enhanced in DLBCL compared with rLN, which was mainly attributed to the strong interactions of B cell cluster with NK sub-cluster. It is well known that SEs interact with transcription factors to promote target genes transcription [21]. We wondered whether the expression of CCL3, CD70 and MIF is regulated by SEs in DLBCL cells. We found that DOHH2, HBL1 and NU-DHL1 cells had SEs only at the *CD70* locus but not at the *MIF* or *CCL3* locus. Furthermore, we demonstrated that *CD70* was a SE-controlled gene in DOHH2 cells and its expression was driven by the transcription factor ZZZ3.

CD70 is a member of the tumor necrosis factor (TNF) ligand family [56]. It has been reported that CD70 expression in non-Hodgkin lymphoma cells upregulates the proportion of Foxp3+CD4^+^CD25- T cells and inhibits the proliferation of infiltrating CD8^+^ T cells, thereby promoting an immunosuppressive microenvironment [57]. Co-inhibition of CD70 and PD-L1 rescued T cell depletion and effectively inhibited lymphoma growth *in vivo* [58]. However, the role of CD70 in regulating the interaction between DLBCL cells and NK cells remains unclear.

ZZZ3 (zinc finger ZZ-type containing 3), a core subunit of the ATAC complex, is required for ATAC complex-mediated maintenance of histone acetylation and gene activation [59]. However, we have not found any studies of ZZZ3 in regulating DLBCL progression. In this study, we found that high ZZZ3 expression predicts a poor OS of DLBCL patients. Importantly, we found that under co-culture of DOHH2 cells and NK92 cells, silencing CD70 in DOHH2 cells significantly promoted the proliferation of NK92 cells and LDH release from DOHH2 cells, which could be partially counteracted by ZZZ3 overexpression in DOHH2 cells. LDH is a ubiquitous intracellular enzyme that is released outside the cells when cells die [60]. LDH release is a key indicator of lytic cell death [60]. Thus, our results suggested that ZZZ3/CD70 axis in DLBCL cells promoted their escape from infiltrating NK cell cytotoxicity and inhibited the proliferation of infiltrating NK cells.

Moreover, mounting studies have provided that aberrant expression of PD-L1 of lymphoma cells is critical for mediating tumor immune evasion. Blocking the interaction between PD-L1 and PD1 could restore the anti-tumor immune response [11, 61, 62]. In this study, we found that both *CD70* and *CD274* (PD-L1 encoding gene) were expressed in Bcell_2 sub-cluster, and *PDCD1* (PD1 encoding gene) was expressed in NK cell sub-cluster, suggesting that targeting CD70 and PD-L1 simultaneously may effectively enhance the killing of DLBCL cells by NK cells. To verify this hypothesis, we demonstrated that silencing CD70 in DOHH2 cells combined with PD-L1 blockade significantly promoted killing of DLBCL cells by NK cells. These results suggested that CD70 plays a key role in the evasion of DLBCL cells from NK cell killing. Targeting CD70 in combination with anti-PD-L1 therapy could be a promising strategy for DLBCL treatment.

## Conclusion

*CD70* was an SE-controlled gene that was driven by ZZZ3 for transcription in DLBCL cells. The ZZZ3/CD70 axis in DLBCL cells inhibited infiltrating NK cell killing and proliferation, thereby promoting immune evasion of DLBCL cells. The ZZZ3/CD70 axis has the potential to be a novel immunotherapy target for DLBCL. Targeting ZZZ3/CD70 axis combined with PD-L1 blockade is expected to be a promising immunotherapeutic strategy for the treatment of DLBCL.

## Data Availability

The raw data supporting the conclusions of this article will be made available by the authors, without undue reservation.

## Footnotes

1
https://heidata.uni-heidelberg.de/

2
http://www.ncbi.nlm.nih.gov/geo/

3
http://bio-bigdata.hrbmu.edu.cn/CellMarker/index.html

4
http://genome.ucsc.edu/

5
http://dbtoolkit.cistrome.org
