# Supplementary material for: Integrated multi-omics profiling reveals the ZZZ3/CD70 axis is a super-enhancer-driven regulator of diffuse large B-cell lymphoma cell-natural killer cell interactions
Source: Exp Biol Med (Maywood). 2024 Sep 23;249:10155. doi: 10.3389/ebm.2024.10155 (PMC11457841; doi:10.3389/ebm.2024.10155)
Supplement: Supplementary file 1 [file DataSheet1.PDF]

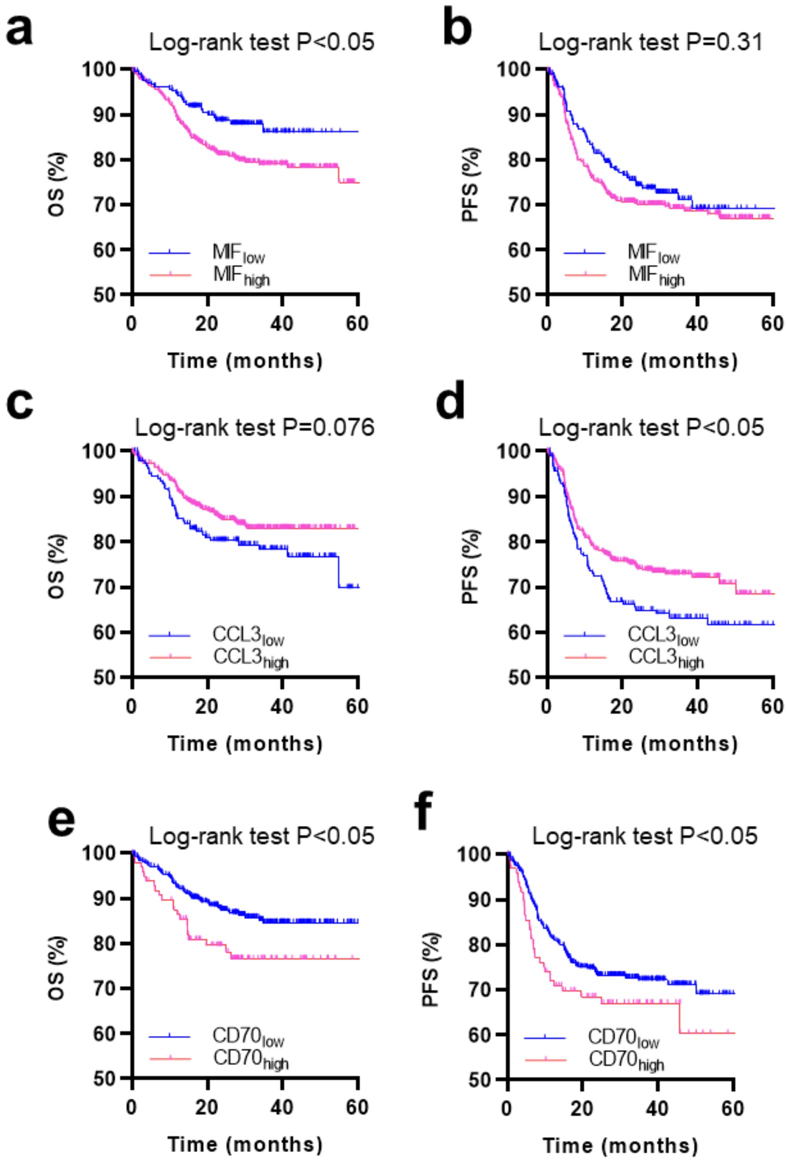

Figure S1 Kaplan-Meier survival curves for overall survival (OS) and progression-free survival (PFS) stratified by MIF (a-b), CCL3 (c-d) and CD70 (e-f) expression levels based on GSE117556 dataset.

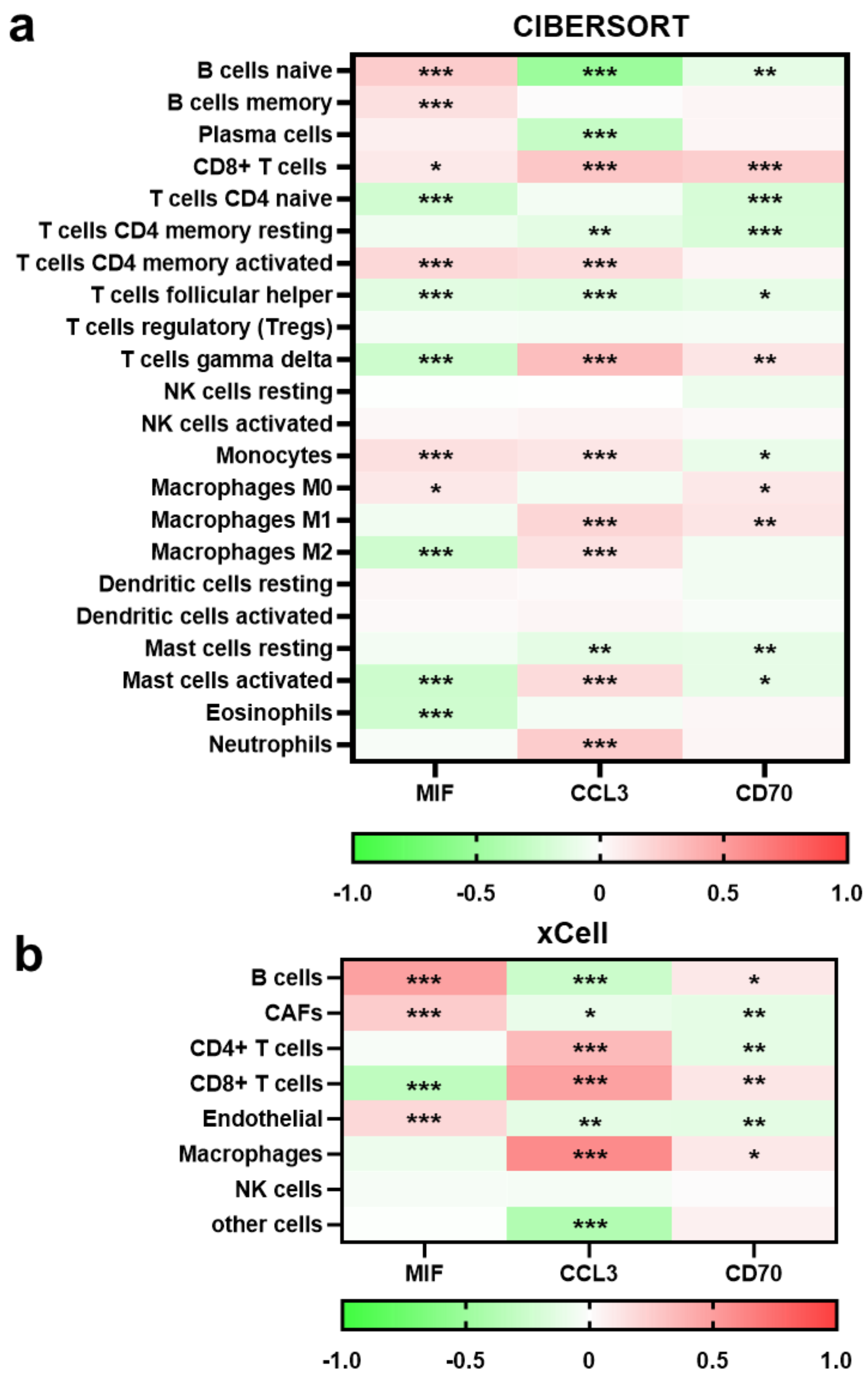

Figure S2 Heatmap of correlations between MIF, CCL3 and CD70 expression levels and CIBERSORT (a) and xCell (b) scores. Red indicates positive correlation, green indicates negative correlation. The deeper the color, the greater the absolute value of the correlation coefficient. *P<0.05, **P<0.01, ***P<0.001.
